# Automated recognition of pain in cats

**DOI:** 10.1038/s41598-022-13348-1

**Published:** 2022-06-10

**Authors:** Marcelo Feighelstein, Ilan Shimshoni, Lauren R. Finka, Stelio P. L. Luna, Daniel S. Mills, Anna Zamansky

**Affiliations:** 1 Information Systems Department, University of Haifa, Haifa, Israel; 2 School of Veterinary Medicine and Science, The University of Nottingham, Nottingham, UK; 3 Department of Veterinary Surgery and Animal Reproduction, School of Veterinary Medicine and Animal Science, São Paulo State University (Unesp), Botucatu, São Paulo Brazil; 4 School of Life Sciences, Joseph Bank Laboratories, University of Lincoln, Lincoln, UK

**Keywords:** Animal behaviour, Computational science, Computer science

## Abstract

Facial expressions in non-human animals are closely linked to their internal affective states, with the majority of empirical work focusing on facial shape changes associated with pain. However, existing tools for facial expression analysis are prone to human subjectivity and bias, and in many cases also require special expertise and training. This paper presents the first comparative study of two different paths towards automatizing pain recognition in facial images of domestic short haired cats (n = 29), captured during ovariohysterectomy at different time points corresponding to varying intensities of pain. One approach is based on convolutional neural networks (ResNet50), while the other—on machine learning models based on geometric landmarks analysis inspired by species specific Facial Action Coding Systems (i.e. catFACS). Both types of approaches reach comparable accuracy of above 72%, indicating their potential usefulness as a basis for automating cat pain detection from images.

## Introduction

Accurate recognition and assessment of pain in animals is crucial for pain management and welfare assessment. Facial expressions are identified as one of the most common and specific indicators of pain in (human and non-human) animals. Since Langford et al^1^ first reported on facial expressions associated with pain in mice, there has been growing interest in tools for assessment of facial expressions linked to pain in a range of non-human animal species. Such tools have been created and validated for various species, including rats^2^, rabbits^3^, horses^4^, pigs^5^, sheep^6^, ferrets^7^ and cats^8^. Optimal pain management in cats presents various challenges due to a lack of well-established management strategies for certain painful conditions^9^, reservations about the adverse effects of analgesics^10^, uncertainty around the reliability and specificity of certain behavioural indicators of pain^11^ and a limited ability of humans to accurately interpret facial expressions associated with negative affect in this species^12^. Such factors may also contribute to cats tending to be prescribed less analgesic drugs by veterinarians compared with dogs^13,14^.

The gold standard of objectively assessing facial expressions in human emotion research is the Facial Action Coding System^15^ (FACS), which measures the individual movements or ‘Action Units’ (AUs) among the muscles of the face, assigning codes to activity of individual muscles or groups of muscles. FACS has recently been adapted for several non-human species, including several non-human primates^16^, dogs^17^ and cats^18^, aiming also at facilitating cross-species comparison^19^. However, major limitations of FACS are that it requires extensive human training for reliable application, requires dynamic change in the face, for which it can be difficult to clearly identify an apex, is time consuming and in some cases can be costly to undertake and may be prone to human error or bias^20^.

A simplified method, applied mainly in the context of pain, are grimace scales. They are species-specific and have been developed for several species, such as mice^1^, rats^2^, cows^21^, lambs^22^ and cats^23^. While grimace scale based tools are much simpler to learn and use than animalFACS-based methods, they remain subject to bias due to the use of human raters. Furthermore, they do not readily support the exploration of facial lateralisation, even though previous evidence would suggest this may be important in relation to the facial expression of affective states such as pain^24,25^. Finally, their development is largely underpinned by a priori assumptions about the nature of facial shape changes occurring in relation to pain, which are subsequently quantified using Action Units based on human (rather than species specific) systems (see Finka et al.^26^).

To overcome these limitations, Finka and colleagues^26^ developed a method based on geometric landmarks to identify and quantify facial shape change associated with pain in domestic short-haired cats. These landmarks were based on both the anatomy of cat facial musculature, and the range of facial expressions generated as a result of facial action units (catFACS^18^. The method takes into account individual differences, species-specific facial anatomy, and postural effects. Facial images of 29 cats were captured at four different time points during ovariohysterectomy, corresponding to varying intensities of pain. Images were then annotated using 48 landmarks specifically chosen for their relationship with underlying musculature, and relevance to cat-specific facial Action Units (catFACS). Principal Components (PCs) were extracted to identify key sources of facial shape variation, relative to pain intensity. A significant relationship was found between PC scores and a well-validated composite measure of post-operative pain in cats, UNESP-Botucatu MCPS tool^27^. This demonstrates good convergent validity between the geometric face model, and other well validated metrics of pain detection. This further indicates these landmarks may contain a signal sufficient for machine pain classification, and thus provide a point of departure for our exploration of automatic, objective detection of pain in cats.

Automated facial analysis is a vibrant field in human emotion research. Numerous commercial software tools for automated facial coding are available, some of which can automatically detect FACS Action Units, e.g., FaceReader by Noldus^28^, and Affdex^29^. Some researchers consider automated tools to have greater objectivity and reliability than manual coding, eliminating subjectivity and bias^30,31^. In the context of pain, numerous works have addressed facial expression assessment in humans, and especially in infants. The Prkachin and Solomon Pain Intensity score^32^ is a useful tool for pain intensity scoring based on FACS AUs. Prkachin^33^ provides an extensive review of the history of pain assessment via facial expressions, also emphasizing the future potential of automated analysis. A review of state of the art methods of automated pain analysis in infants is provided by Zamzmi and colleagues^34^, where facial expressions are identified as one of the most common and specific indicators of pain. Facial expression of pain is defined as the movements and distortions in facial muscles associated with a painful stimulus. The facial movements associated with pain in infants include deepening of the nasolabial furrow, brow lowering, narrowed eyes, chin quiver and more^35^. Methods proposed for extracting infants’ pain-relevant features from images can be divided into those focusing on static images, and those focusing on the temporal analysis of facial expression in videos, applying motion-based or FACS-based analysis. In our study, the former approach is taken.

In non-human animals, however, automation of facial analysis in relation to the detection of affective states such as pain remains underexplored. Hummel and colleagues^36^ highlight some important challenges in addressing automated pain recognition in animals, which can also be generalized to emotion recognition in general. First of all, much less data is available, compared to the vast amounts of data in the human domain. Furthermore, particularly in the case of domesticated species selected for their aesthetic features, there may be comparatively much greater variation in their facial morphology, making population-level assessments problematic, due to the potential for pain-like facial features to be more present/absent in certain breeds at baseline^26^. Finally, and perhaps most crucially - there is no verbal basis for establishing ground truth, whereas in humans, self-reporting is commonly used. This complicates data collection protocols for non-human animals, requiring conditions where the induction of a particular affective state and its intensity must be closely controlled and regulated, or requires rating by human experts, potentially introducing bias and a circular logical flaw relating to human expectation for objective validity.

Automated emotion and pain recognition in animals has thus far only been addressed in a few species. Sotocinal and colleagues^2^ introduced a partially automated approach for supporting pain level assessment on rats, which served as a prescreening tool selecting frames for manual assessment. Tuttle et al.^37^ use deep learning to perform automatic facial Action Unit detection in mice, to score for pain. The single-frame InceptionV3 CNN is trained to detect Action Units, which are counted according to a grimace scale for pain detection. The model reached accuracy of 94% at assessing the presence of pain in mice across different experimental assays. Andresen et al.^38^ further presented accuracy of up to 99% for the recognition of the absence or presence of post-surgical and/or post-anesthetic effects as part of the development of a fully automated system for the surveillance of post-surgical and post-anesthetic effects in mice. Mahmoud et al.^39^ proposed a pipeline for automation of pain level assessment in sheep, based on detection of 9 pain related AUs. They are detected through the successive steps of facial detection, landmark detection, feature extraction and classification with an SVM using histogram-related features (HOG). Using a dataset of 480 images of sheep, an average classification accuracy of 67% in AUs was reached. Lencioni et al.^40^ trained a convolutional neural network for pain assessment in horses undergoing castration. The study assessed facial expressions of 7 horses, recorded from the top of the feeder station, capturing images at 4 distinct times daily for two days before and four days after surgical castration. The trained model reached an overall accuracy of 75.8% while classifying pain on three levels: not present, moderately present, and obviously present; while classifying between two categories (pain not present and pain present) the overall accuracy reached 88.3%.

Hummel et al.^36^ applied a landmark-based approach to pain recognition in horses, exploring also the transfer of the method to donkeys. The used dataset consisted of 1854 images of horse heads and 531 images of donkey heads, annotated by vet experts who scored the pain level of the horses and donkeys based on six pain features derived from equine grimace scales^4^. The images were annotated with 44-54 landmarks (depending on the head pose; additional landmarks described the contour of the face. The landmarks were used for cropping four Regions of Interest (ROIs): the eyes, ears, nostrils, and the mouth. Various histogram-related features commonly used in image processing were then extracted, such as HOG, local binary patterns (LBP), scale-invariant feature transform (SIFT) and features created by a VGG16 deep neural network model. A fusion of different classifiers based on different types of these features was shown to improve results. The study achieved 0.51-0.88 F1 score on pain estimation on tilted poses (with different face regions), and 0.53-0.87 after decision fusion of classifiers based on different features. Neither automatic landmarking, nor pain estimation were found directly transferable to donkeys. Morozov et al.^41^ developed and implemented a prototype system for automatic MaqFACS coding, using a dataset which included 53 videos from 5 Rhesus macaques capturing frontal views of head-fixed individuals; the video-frames were manually coded for the AUs present in each frame. The system was trained to classify six MacFACS AUs reaching average 89% accuracy. As opposed to the works mentioned above, using still images, Broome and colleagues^42^ considered the dynamics (temporal properties) of pain expressions by training a deep learning neural network for the task of distinguishing induced pain from non-pain in videos of horses. The dataset consisted of 9 hours and 45 minutes of video across six horse subjects (out of which 3 hours and 41 minutes are labeled as pain and 6 hours and 3 minutes as non-pain), labeled according to the presence of pain induction. The study concluded that spatio-temporal unfolding of behavior, and specifically sequentiality, is crucial for pain recognition in horses, since horses do not convey a single straightforward face to show that they are in pain, the way humans can. The classifier reached accuracy of around 75% in recognizing (induced) pain, performing better compared to manual scoring by four veterinary experts, who only reached around 58% accuracy in recognizing pain. Poorer performance of human experts as compared to machine classification of horse pain was further reinforced in^43^ in the context of assessing low grade orthopedic horse pain. The authors further showed that a model trained solely on a dataset of horses with acute experimental pain (where labeling is less ambiguous) can aid recognition of the more subtle displays of orthopedic pain. Another notable method for equine pain classification was used in^44^, where latent pose representation is extracted from multi-view surveillance video footage of horses with induced orthopaedic pain, reaching accuracy of above 60%. The disentangled pose representation is used to ensure that pain is learned from horse body language alone.

A recent review by Andersen et al.^45^ provided a detailed comparison in the context of horse pain detection between approaches based on automatic detection of AUs, and deep learning models that are trained on raw videos of horses with known true pain status, both of which present promising results. Blumrosen and colleagues^46^ proposed a different approach to overcome the difficulties in creating human-annotated datasets of facial expressions in non-human primates (NHP). The proposed framework learns the statistical features of each NHP’s facial expressions with minimal prior assumptions, using a two layers unsupervised clustering algorithm. A preliminary evaluation of the framework was performed using video recordings of 12 mins of one head-fixed rhesus macaque and four facial expressions of neutral, lip smacking, chewing, and random mouth opening. The clips were tagged based on the MacFACS by three independent unbiased experts in NHP behavior with a majority voting protocol. The machine’s results were about 82% accurate compared to the rating by the human experts.

Thus automated pain recognition has so far only been addressed for rodents, horses and sheep. Similarly to the human domain, the majority of efforts focuses on deep learning approaches (e.g.,^37,40^), but also classical machine learning models with hand-crafted features which offer more explainability have been considered (e.g.,^36,39^). In this paper we investigate both of these directions in the context of cat pain. The lack of benchmarking datasets makes it difficult, and sometimes infeasible to compare the different approaches in terms of accuracy, as the datasets differ in numerous dimensions with respect to data acquisition and ground truth annotation: naturalistic vs. controlled setting, induced vs. natural emotional state/pain, use of fixation of the animal, angle of footage, degree of agreement between annotators (if relevant) and many other factors. Thus, e.g., the 99% accuracy achieved in^38^ of pain recognition in laboratory mice cannot be straightforwardly compared to the estimation of pain level with accuracy of 67% accuracy in sheep using footage obtained in the naturalistic setting of a farm.

This paper is the first to explore automated recognition of pain in cats, classifying facial images of cats into pain/no pain categories. We compare two different approaches along the lines of two main research streams in the literature: the first, following the approach taken in Finka et al.^26^, uses 48 manually annotated geometric facial landmarks, positioned relative to underlying facial musculature, as an input to the model, and applies multi-region vectorization based on these landmarks. This approach has only been studied so far in the context of human facial analysis^47^, and has not been applied to animal facial images. The second approach we consider does not rely on landmarks: it uses a deep learning network which takes as input raw images of cat faces. Such an approach requires less annotation efforts, yet the model is ‘black-box’ and lacks explainability, i.e., it is less understandable for humans how the network makes its decisions.

## Results

### Dataset

We used the dataset generated as part of a previous study by Finka et al.^26^. The raw data comprised of footage from 29 healthy mixed breed (domestic short hair) female cats (2.8 +/-0.5 kg; 14.1 +/- 5.2 months) undergoing ovariohysterectomy as described in Brondani et al.^27^. Cats were recorded at different time points corresponding to varying intensities of pain: Pre-surgery (between 18-24 hours during the preoperative period), 1 hour post-surgery (between 30 minutes and 1 hour after the end of surgery, and prior to administration of additional analgesics), and Post-rescue analgesia (approximately four hours after postoperative analgesia).

**Figure 1:**
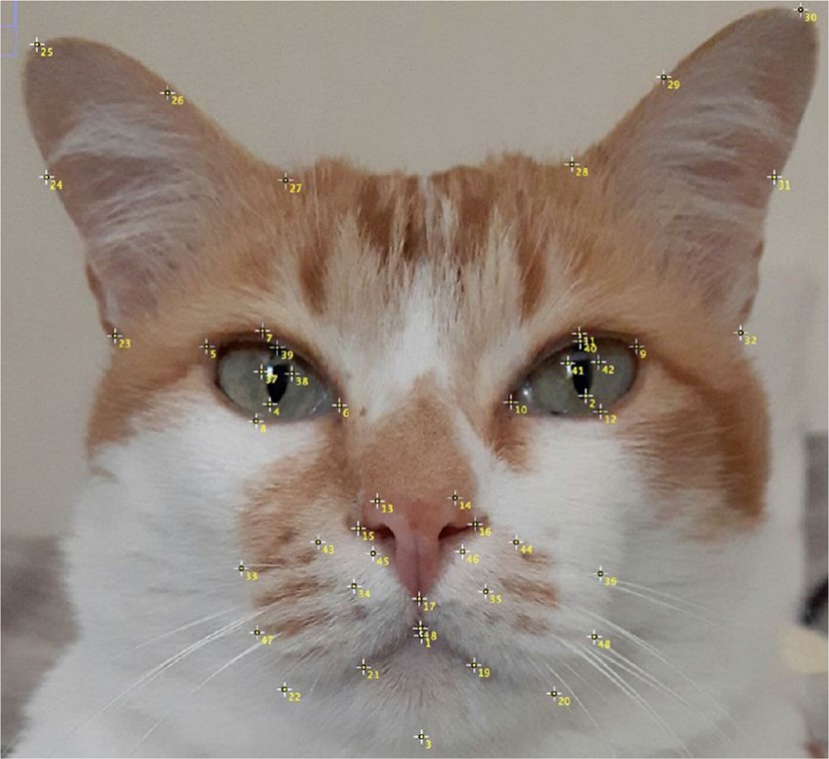
Mirror image of cat’s face, depicting placement of the 48 facial landmarks. Landmarks appear contralateral to their origin, as they would when directly observing the cat’s face.

The images were annotated with 48 landmarks, landmarks were specifically chosen for their relationship with underlying musculature, and relevance to cat-specific facial Action Units (catFACS^18^), were added. For the specific location of each landmark, see Fig. 1.

For our final dataset we selected images from 26 cat individuals with 232 images of ‘No Pain’ (Pre Surgery stage and Post-rescue analgesia stage), and 232 images of ‘Pain’ (1 hour post surgery), overall 464 balanced images. We discarded images of three cats for having only Pain or No Pain class images. The dataset, including images and landmark annotations, is publically available at https://gitlab.com/is-annazam/automated-recognition-of-pain-in-cats.

### Classification results

Table 1 presents a comparison of the different obtained models in the two approaches of DL (deep learning approach) and LDM (landmark-based approach). For DL, a pre-trained Resnet50^48^ network architecture was used. For LDM, we used the Multilayer Perceptron (MLP) neural network^49^ with three hidden layers. In each of the approaches, we performed training with and without data augmentation and cat face alignment to test the effect of each of these steps on performance.

To compare the models, we use the following metrics which are standardly used in the context of machine learning models performance evaluation (see, e.g., Lencioni et al^40^).

Total is the set of all images. The set of True Positives contains images, belonging to class ‘Pain’ (i.e. the images extracted from video footage of cats post-operatively, prior to rescue analgesia, considered to be in pain), which have been correctly classified by the model as ‘Pain’. The set of True Negatives contains images, belonging to class ‘No Pain’ (i.e. the images extracted from video footage of the same cats pre-operatively, considered to not be in pain), which have been correctly classified by the model as ‘No Pain’. The set of False Positives contains images, belonging to class Pain (as defined above), which have been incorrectly classified by the model as No Pain. The set of False Negatives contains images, belonging to class No Pain (as defined above), which have been incorrectly classified by the model as Pain.

Accuracy, indicating the overall efficacy of the model, is calculated by:

$${\text{Accuracy}}\,{\text{ = }}\,{\text{(sizeof(True Positives) + sizeof(True Negatives)) / sizeof(Total) }}$$

Precision, indicating whether the image was classified in the correct class, is calculated by:

$${\text{Precision = sizeof(True Positives)/ (sizeof(True Positives + sizeof(False Positives)) }}$$

Recall, measuring the ability of the classifier to identify all the correct data for each class, is calculated by:

$${\text{Recall = sizeof(True Positives)/ (sizeof(True}}\;{\text{Positives + sizeof(False Negatives))}}$$

As a validation method^50^, we use leave-one-subject-out cross validation with no subject overlap. Due to the relatively low numbers of cats (n=26) and of samples (N = 232*2) in the dataset, following the stricter method is more appropriate^38,42^. In our case this means that we repeatedly train on 19 subjects, validate on 6 and test on the remaining subject; Table 1 presents the aggregated average result. By separating the subjects used for training, validation and testing respectively, we enforce generalization to unseen subjects and ensure that no specific features of an individual are used for classification.

**Table 1:** Classification performance comparison.

Approach	Align	Augment	Accuracy	Precision	Recall
DL	Yes	No	0.7239 (+− 0.1837 )	0.7526 (+− 0.2139 )	0.7353 (+− 0.3215 )
DL	Yes	Yes	0.7051 (+− 0.1855)	0.7725 (+− 0.2385 )	0.6853 (+− 0.3195)
DL	No	No	0.7360 (+− 0.1782)	0.8186 (+− 0.2045)	0.7010 (+− 0.2889 )
DL	No	Yes	0.7344 (+− 0.1780 )	0.84512 (+− 0.1948 )	0.6636 (+− 0.3614 )
LDM	Yes	No	0.7196 (+− 0.1464)	0.7441 (+− 0.1600)	0.7457 (+− 0.1943)
LDM	Yes	Yes	0.7239 (+− 0.1290)	0.7315 (+− 0.1451)	0.7512 (+− 0.1955)
LDM	No	No	0.6747 (+− 0.1151)	0.7056 (+− 0.1442)	0.6892 (+− 0.2639)
LDM	No	Yes	0.6805 (+− 0.1087)	0.6807 (+− 0.1103)	0.6933 (+− 0.2278)

## Discussion

Overall, both DL and LDM reached roughly similar accuracy of above 72%. As mentioned above, comparing accuracy results between studies in the domain of automated approaches for animal emotion/pain recognition is challenging due to the critical differences in data acquisition and annotation, but even more importantly - in terms of the ways of measuring performance of models. One important point in the way performance was measured here is subject-exclusive validation, i.e., ensuring there is no subject overlap between training and testing sets. This is crucial for ensuring that individual specific features are not learnt by the classification model. Such technique of validation has been used only in a few works for recognizing pain: Broome et al.^42^ reached 75.4% for horses, Rashid et al.^44^ reached 60.9% for horses and Andresen et al.^38^ reached 89.8% for laboratory mice. While the latter high accuracy can be attributed to better ability to capture quality facial images of laboratory mice (e.g., better lightening control) as opposed to larger animals in naturalistic settings, our results outperform those of^44^ and are comparable to those of^42^.

This study is the first to apply automated approaches for pain recognition in feline species. However, the study population was limited to young, intact (at the point of recruitment) adult female cats of a single breed type (as described in the Methods section above). For a proof of concept stage, using a relatively demographically homogenous population of domestic cats is advantageous. This is given the degree to which breed differences can be associated with drastic alterations in the underlying facial morphology of individuals and potentially the degree to which pain-linked expressions (based on LDM methods) are displayed^51^. However, given that factors such as breed^51,52^, age^53^ and potentially their interaction^54^, as well as sex^55^ and particularly neuter status in adult males (e.g.^56^), may all affect aspects of craniofacial morphology in this species, future work should aim to assess the degree to which such cat-characteristics may impact on model performance, with the aim to increase model generalisability and robustness. However, for a proof of concept stage for the development of AI methods to detect pain via cat facial expressions, using a relatively demographically homogenous population of domestic cats at a proof of concept stage for the development of AI methods to detect pain via cat facial expressions is actually advantageous. This is because breed differences can be associated with drastic alterations in the underlying facial morphology of individuals^52^ and potentially the degree to which pain-linked expressions are displayed^51^.

Secondly, domestic cats are closely related to wild, primarily asocial progenitors^57,58^ and are not considered to have well-developed or highly selected capacities for social communication via their facial expressions, as may be the case in domestic dogs for example^59^. While pain-linked variation in facial expressions in domestic cats are established^8,23^, these differences are potentially morphologically subtle at the population level^26^. Given that variations in cat behaviour and posture may also provide useful in the detection of pain in this species^23,27,60^ and also demonstrate convergent validity with facial-based systems of pain assessment^23,26^.

However, it should be noted that body posture and movement may also provide useful information about pain and the two show convergent validity. With a high correlation (Spearman rank correlation coefficient of 0.86) between the Feline Grimace Scale and the Glasgow composite measure pain scale reported for acute pain in cats^23^. Given that variations in cat behaviour and posture may also provide useful information in the detection of pain in this species^27,60^, future of optimization of AI based methods for cat pain assessment should aim to combine both facial and postural elements.

The results provide an indication that both of the approaches desribed here are potentially useful as a basis for automated pain recognition in cats. However, further methodological improvements are necessary if automated approaches are to be relied upon to support pain assessment and its effective treatment for individual cat patients, in real-life contexts. In particular, given the potentially high welfare costs to animals associated with their undetected or undertreated pain, initially prioritizing refinements to DL approaches which increase their ability to correctly identify cases of ‘true positives’ may be most beneficial from an animal welfare perspective. An important part of this optimization process is to also understand the contributing characteristics of misclassified individuals. A suggested, further work which incorporates additional behavioral information during the classification process and includes larger and more heterogenous study populations may help to achieve this.

None of the approaches were improved by data augmentation (noise injection); the LDM approach performed better coupled with face alignment, thus we conclude the inclusion of this pre-processing step is important for optimizing performance of landmark methods which use vectorization. This difference could be due to the deeper nature of the ResNet architecture, allowing it to better deal with rotation, as opposed to the shallower network used in LDM. In comparison to the works of Mahmoud et al.^39^ on sheep, and Dalla Costa et al.^4^ on horses and donkeys, using facial landmarks. we also use landmarks to localize facial areas of interest. However, while these works extract histogram-related features from these areas, we use the landmarks for vectorization following the approach of Qiu and Wan^47^ for human facial analysis. A future research direction is comparing these appoaches in the context of cats.

Observing the conceptual differences between our two studied approaches is crucial for the interpretation of the obtained results. First of all, while the DL approach gets information from the raw images, the LDM approach uses a much more limited representation of 48 landmarks. Comparable accuracy between the approaches implies that the results support the conclusion that the subset of information contained in the suggested 48 geometrical facial landmarks is sufficient for pain classification, with an accuracy comparable to using raw images which contain more visual information. Therefore, the landmarks suggested by Finka et al.^26^ are confirmed as a useful method to identify and quantify changes in facial shape associated with pain in a non-human animal model. Secondly, the features used by the machine learning models in the two approaches are of a different nature: in the DL approach, feature selection is done automatically, while in the LDM approach the features are the vectors obtained from the 48 landmarks objectively chosen by humans. At this stage, we can conclude that using Resnet as deep learning backbone does not outperform humans in feature selection. This, however, can possibly be improved by using more sophisticated network architectures and pipelines such transformers^61^.

To summarize, each of the studied approaches has its benefits and pitfalls. The LDM approach assumes the availability of landmark annotation, which is currently a labor-intensive task. Automation of this process requires large annotated datasets for training, yet seems to be feasible, based on successful efforts in the human domain (see Wu and Ji^62^ for a survey of state-of-the-art techniques), as well as the results of Mahmoud et al.^39^ and Dalla Costa et al^4^ in the context of horses and sheep. Thus, an immediate direction for further development of automated pain detection in cat facial features is to automate the process of landmark detection, as well as investigate the relative predictive value or weighting of each individual landmark, in order to determine which are essential for optimal model performance. Moreover, the DL approach was not shown to be sensitive to facial alignment, while for the LDM approach facial alignment yielded higher accuracy, therefore we conclude it is recommended as a preprocessing step in landmark-based approaches. On the other hand, the LDM approach is based on lighter models which require less computational resources to run, and may be more geared toward implementations into mobile environments such as a dedicated application that could run from the average smartphone. Moreover, the DL approach is ‘black-box’ reasoning, which does not borrow itself easily for explaining the classification decisions in human-understandable terms (see, e.g.,^63^); in clinical contexts future research into explainability aspects is required. One immediate direction for future research is looking into visualization of layers of the networks, using different techniques such as heatmaps, attention maps, t-sne visualizations and more.

This work not only demonstrates the intellectual value of multidisciplinary collaboration between biologists and computer scientists, but especially the increasing value of machine learning techniques within the biological sciences. This includes, for example in the current case, both the obvious applied value of the potential to develop automated systems to assist in the assessment of pain in animals for both diagnostic and management purposes by both veterinarians and welfare scientists, but also its potential to address more fundamental issues, such as evidencing the existence of pain (and potentially other emotional states) in non-human species and the efficacy of new psychotherapeutic interventions. Indeed we can also foresee a time when using methods such as those applied here to diverse species it might be possible to construct phylogenies mapping the expression of responses to painful stimuli, which might usefully inform the fundamental philosophical debate on the minimum extent to which certain forms of sentience should be considered to exist in other species.

## Methods

The dataset relating to the cats used for this study was collected previously under the following ethical approvals of the Institutional Animal Research Ethical Committee of the FMVZ-UNESP-Botucatu (protocol number of 20/2008) and the University of Lincoln, (UID: CoSREC252) as per Finka et al^26^. The current protocol using this data was reviewed by the Ethical Committee of the University of Haifa and no further approval was required. All experiments were performed in accordance with relevant guidelines and regulations.

### Cat facial alignment

Face alignment^64^ is the process that deforms different face images such that their semantic facial landmarks/regions are spatially aligned. It reduces the geometric variations of faces and eases the face processing. It is widely used to boost the performance of face recognition, actually serving as an inseparable step. One of the existing face recognition approaches^65,66^ first localizes facial landmarks via supervised learning and applies a hand-crafted deformation function (e.g. affine or projection transformations) to deform the image to match a pre-defined template. Adapting this method to cat faces, given a set of facial landmarks (the input coordinates) we transform the images to an output coordinate space so that the cats’ face is (1) in the image center, (2) rotated so the line formed by the eyes is horizontal (i.e., eye landmarks have the same y-coordinates), and (3) scaled so that the size of the faces are approximately identical across the dataset.

The alignment process starts by calculating the center points of each eye (middle of landmarks 37 and 38 for left eye and middle of landmarks 41 and 42 for right eye, see Fig. 1). Given the eye centers, we can compute the coordinate differences and obtain angle of rotation between the eyes.

The left eye centers of all images were located on a fixed point of the image (we set this point on the 0.4 of the output image width and 0.4 of output image height from upper-left corner). We also predetermined that all output images had a width of 1000 pixels and height of 1000 pixels. Having those parameters, we determined the location of the right eye assuming that the new location of the right eye should be equidistant from the right edge of the image as the corresponding left eye x-coordinate is from its left edge. The scaling factor between the input image and the output image is calculated by taking the ratio of the distance between the eyes in the current image to the distance between eyes in the desired image. Having the center point between both eyes, the rotation angle and the scale, we perform a 2-dimensional translation and a rotation transformation of the input image obtaining the output image. The landmarks of the output images are obtained transforming the input landmarks in the same way the images were transformed.

**Figure 2:**
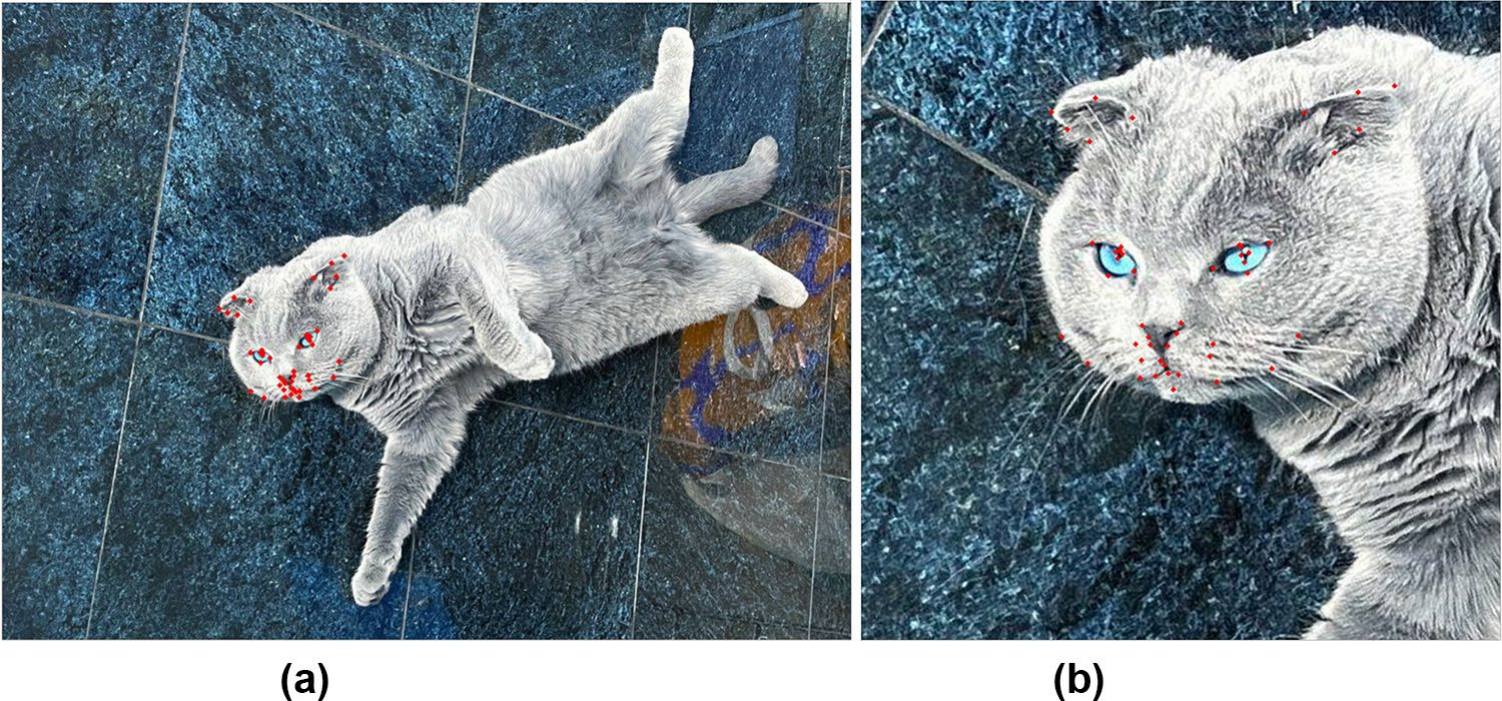
Image before and after alignment.

As an example, we can see on Fig. 2a an original image in the dataset and its aligned image on Fig. 2b.

### Approach 1: landmark-based

The first model we developed takes as input multi-region vectors, a concept adapted from the approach in Qiu at al.^47^ where human facial analysis is performed based on multi-region vectors, formed by landmarks divided into different face regions. We adapt the notion of multi-region vectors of Qiu at al.^47^ to the context of cat faces based on the Feline Grimace Scale^23^ as follows.

**Figure 3:**
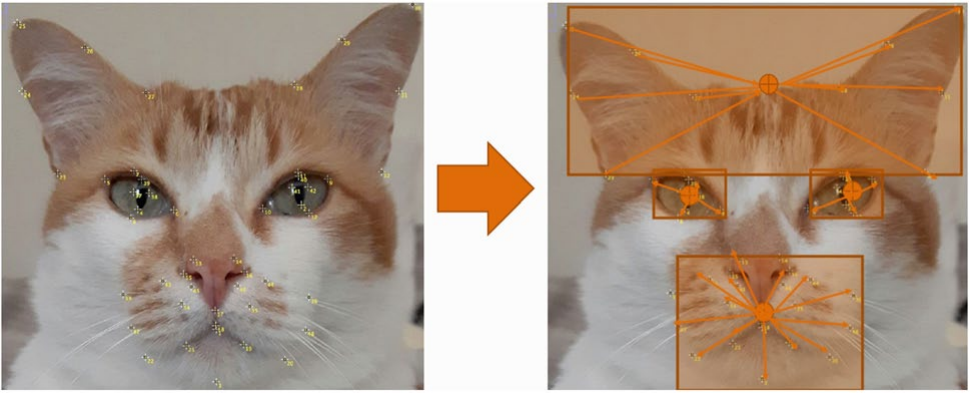
Landmark transformation into vectors, and division into four regions of interest according to the Felice Grimace Scale.

We define four regions of interest on cat faces: left eye region (defined by landmarks 4, 5, 6, 7, 8, 37, 38, 39, with central point on the median of landmarks 37 and 38); right eye region (defined by landmarks 2, 9, 10, 11, 12, 40, 41, 42 with central point on the median of landmarks 41 and 42); nose, mouth and whiskers region (defined by landmarks 1, 3, 13, 14, 15, 16, 17, 18, 19, 20, 21, 22, 33, 34, 35, 36, 43, 44, 45, 46, 47, 48 with central point on landmark 17); and forehead region (defined by landmarks 23, 24, 25, 26, 27, 28, 29, 30, 31, 32 with central point on the median of landmarks 27 and 28). Figure 3 demonstrates the division.

Based on these four regions, all landmarks are then normalized according to the center of their corresponding regions, creating multi-region vectors.

#### Preprocessing pipeline

**Figure 4:**
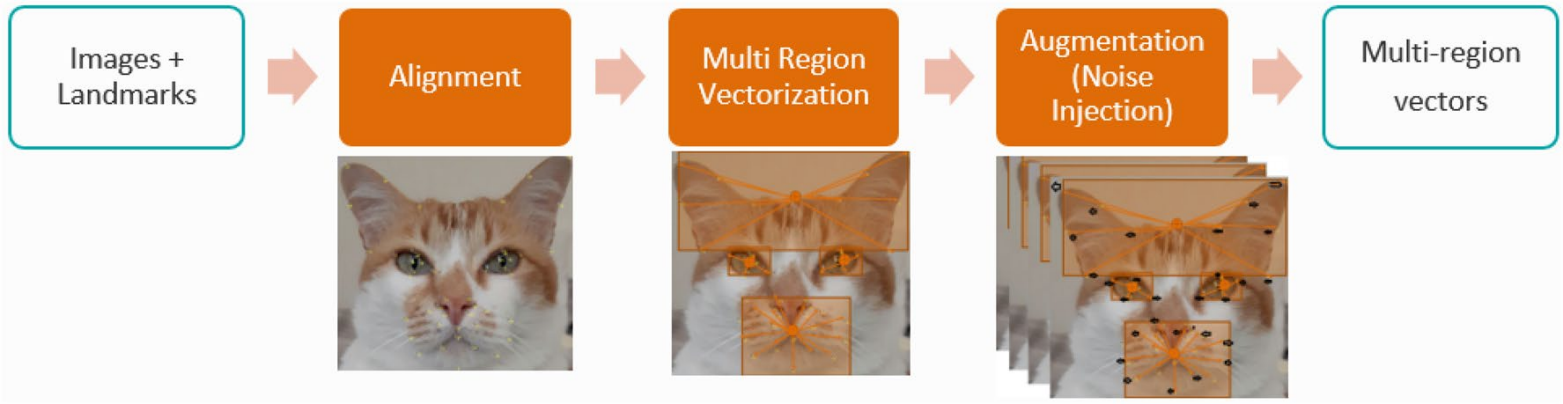
Preprocessing pipeline for model 1.

The first step is facial alignment, which centers the landmarks, and is performed as described in the previous section. The second step is the vector creation, based on the four regions described above. The last step is data augmentation, at which we generate new landmark samples by noise injection^67,68^. Noise injection generates new images by adding certain amount of noise to the training dataset that may lead to a better generalization error and fault tolerance by enhancing the learning capability. Noise injection results in creating new samples multiplying some random values to original samples (i.e. in this case XY coordinates obtained from annotated facial landmarks) following some distribution. In our work, we use noise generated from a normal distribution taking mean as 1, and standard deviation as 0.0005. A new sample is then generated by multiplying every coordinate of the landmarks of the original dataset by a sampled value of normal distributions of this type. The preprocessing pipeline, taking as input images annotated with 48 landmarks, and producing a set of multi-region vectors which are then fed to the model, is described on Fig. 4.

#### Model training

Similarly to^47^, we a use a network compound of an Input layer containing 96 neurons (one for each x and y coordinate obtained via the 48 landmarks), across a 3-hidden layers Multi Layer Perceptron neural network (MLP). The first layer contains 100 neurons using ReLu activation, the second layer 100 neurons using ReLu activation, and the third layer 500 neurons using Relu Activation. An additional output layer of 2 neurons using Softmax activation was also used. The model is trained during 10 epochs optimizing a cross-entropy loss function using Adam method^69^ with learning rate equal to 0.01 with batch size 32. On each epoch, training set is normalized using standard scaling, and augmented. We chose the model that achieved the best (minimal) validation loss.

### Approach 2: deep learning

The second approach we study is a deep learning model, the input of which are raw images (without landmarks).

#### Preprocessing pipeline

**Figure 5:**
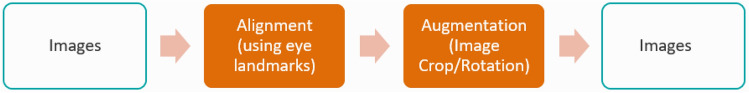
Preprocessing pipeline for model 2.

Similarly to the first approach, we use the same cat face alignment technique. In case augmentation method is applied, but new samples are not generated; instead three actions are applied to each sample on the dataset in order to improve model generalization capability: a crop of a random portion of the image and resizing of it to a given size, a random horizontally flip with a 50% probability and a random rotation with an angle in the range between -90 and 90 degrees. The pipeline is shown on Fig. 5.

#### Model training

 We use pre-trained Resnet50 model provided in the Pytorch package for Python using ImageNet weights without its head. On top of the last layer, we add a new sub network compound of the following layers: a Linear layer with 512 cells with ReLu activation, followed by a Dropout layer of 50%, a Linear layer of 256 cells with ReLu activation, followed by a Dropout layer of 40%, a Linear layer with 128 cells with ReLu activation followed by a Dropout layer of 30%, a Linear layer with 64 cells with ReLu activation followed by a Dropout layer of 25%, ending with a Linear layer of 2 cells for Pain level categorization (i.e. “No Pain” and “Pain” labels). We fine tune this network, training the network during 200 epochs while freezing only batch normalization layers, using batch size of 64, cross-entropy loss function and optimizing the loss using Adam method with a learning rate of 0.0005. We chose the model that achieved the best (maximal) validation accuracy.

## Data Availability

The dataset in this paper is available upon request.
